# A case of veno-occlusive disease following liver transplantation

**DOI:** 10.3892/etm.2013.1401

**Published:** 2013-11-11

**Authors:** HONG CHEN, XU WANG, TIEYAN FAN, JUN LI, LETIAN WANG, ZHONGYANG SHEN

**Affiliations:** Institute of Organ Transplantation, The General Hospital of Chinese People’s Armed Police Forces, Beijing 100039, P.R. China

**Keywords:** veno-occlusive disease, liver transplantation, tacrolimus, treatment

## Abstract

The present case report describes the diagnosis and treatment of a patient with veno*-*occlusive disease (VOD) following liver transplantation. Combining the clinical data and relevant literature, the study aimed to consider the causes of VOD following liver transplantation, and the pathogenesis, clinical diagnosis and auxiliary examination features of VOD. A 42-year-old man who had a long history of taking traditional Chinese medicine (essential components unknown) underwent an orthotropic liver transplantation on January 14, 2011, due to small venous occlusion disease of the liver. The patient was treated with tacrolimus as an antirejection therapy following the surgery, and gradually developed right upper quadrant pain and fatigue. The examination results were consistent with the diagnostic standards for VOD. Following treatment with methylprednisolone, the patient was treated with alprostadil and Danhong injections. Forty days later, the patient’s total bilirubin (TBIL) level was observed to have decreased significantly, the liver function had returned to normal and the ascites had decreased, but had not completely disappeared. The patient then underwent a transjugular intrahepatic portosystemic shunt (TIPS) procedure, following which the ascites were shown to have completely disappeared.

## Introduction

Hepatic veno-occlusive disease (VOD) is a clinical syndrome characterized by hepatomegaly, ascites, weight gain and jaundice (1–3). VOD has been described in a patient who drank an infusion of a traditional herbal medicine that contained pyrrolizidine alkaloids (4).

Small venous occlusion of the liver was first described in infants with cirrhosis by McFarlane and Branday (5) in 1945, and the underlying liver lesion was recognized as VOD for the first time by Bras *et al*(6) in 1954. Classified small venous occlusion as a VOD was generally accepted from that date. VOD is frequently observed in individuals who have consumed wild plants or herbs containing pyrrolizidine alkaloids, and patients who have undergone hematopoietic stem cell transplantation (HSCT) (7), chemotherapy or radiotherapy; however, VOD following liver transplantation is rare. A patient with VOD following liver transplantation was diagnosed and treated at The Institute of Organ Transplantation (Beijing, China) in 2011. Combining the clinical data and relevant literature, this study aimed to consider the relevant clinical diagnosis, auxiliary examination features, pathological changes and treatment of this case, and the possible causes and pathogenesis of VOD following liver transplantation.

## Case report

### Patient medical history

**Figure 1:**
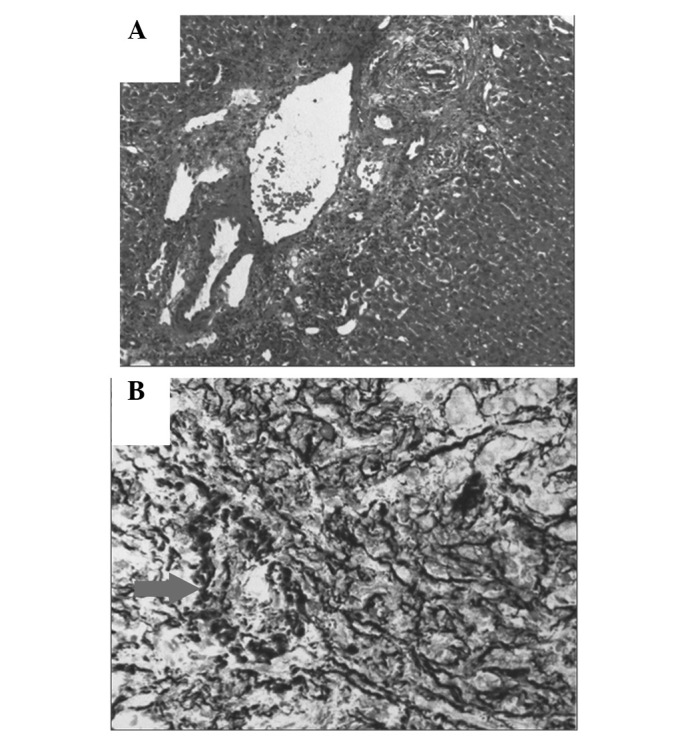
Liver biopsy of the patient. (A) Hematoxylin and eosin (H&E) staining result; magnification, ×5; (B) white mesh fabric plus Masson dyeing with diastase-periodic acid-Schiff (D-PAS) and CK7 antibody (magnification, ×10). The frame indicates multiple lobules with severe congestion around the central veins, while perisinusoidal fibrosis and luminal occlusion of the venules may be observed with the congestion in the center. Multilayer reticular fiber proliferation existed in the small vein cavity.

This study was approved and registered by The Ethics Committee of The General Hospital of Chinese People’s Armed Police Forces in January 2011, the Ethics committee approved relating screening, treatment and data collection. The patient signed a written informed consent form. All works were undertaken following the provisions of the Declaration of Helsinki. A 42-year-old male who had a long history of taking traditional Chinese medicine (essential components unknown) underwent an orthotropic liver transplantation in a local hospital on January 14, 2011, having been diagnosed with small venous occlusion disease of the liver subsequent to splenectomy, combined with incurable ascites. The patient was treated with tacrolimus as an antirejection therapy following the surgery, and developed right upper quadrant pain and fatigue. Routine liver function examinations were conducted which showed that the patient’s alanine transaminase (ALT), aspartate transaminase (AST) and γ-glutamyl transpeptidase (GGT) levels were normal and the alkaline phosphatase (ALP) level was 150–250 IU/l. The total bilirubin (TBIL) level gradually increased to 70 mmol/l and the direct bilirubin (DBIL) level gradually increased to 50 mmol/l, both of which were outside the normal range. The patient’s serum albumin (ALB) level ranged from 25 to 30 g/l and cholinesterase (CHE) level ranged from 500 to 1300 IU/l. The patient’s renal function test indices were: Urea, 20 mmol/l; uric acid (UA), 800–1,000 μmol/l; and creatinine (Cr), 130–140 μmol/l. An ultrasonic B scan of the abdomen showed that there was a low to moderate level of pleural effusion and a large amount of peritoneal effusion; the abdominal computed tomography (CT) and CT angiography (CTA) results showed that anastomotic stenosis was present in the liver artery and inferior vena cava (Fig. 1). The patient received balloon dilatation in the inferior vena cava three times in the local hospital. Following an abdominal CT review, it was revealed that there was no significant stenosis in the inferior vena cava anastomosis; however, the effusion in the patient’s abdomen did not decrease. The patient was then prescribed a maintenance treatment comprising a continual protein supply, diuretic therapy, intermittent hydrothorax fluid extraction (with the maximum volume of 1,000 ml yellow-clear fluid) and catheter drainage of ascites (with a volume of 1,000–1,500 ml/day clear-yellow ascites).

### Admission examination

**Figure 2:**
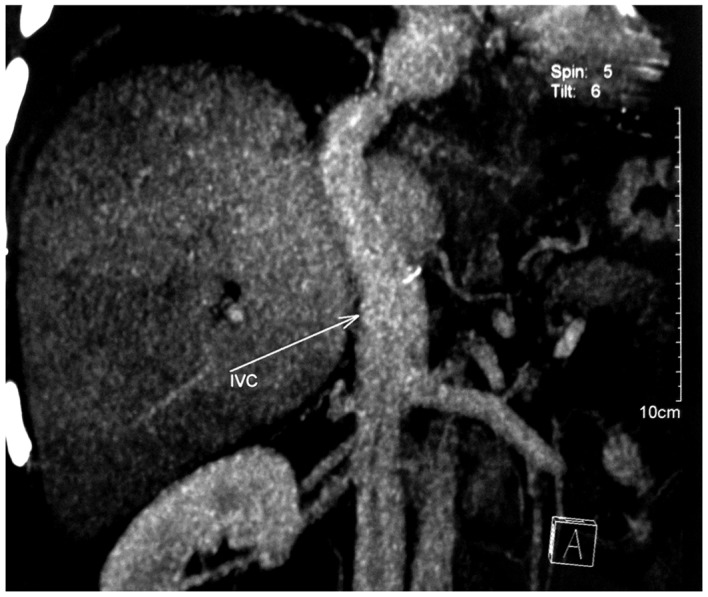
Abdominal computed tomography (CT) and CT angiography (CTA) results showed that no significant stenosis or occlusion existed in the hepatic vein, inferior vena cava (IVC) or portal vein of the patient.

The patient was admitted to The Institute of Organ Transplantation on April 24, 2011. The examination results on admission showed that the patient was weak and in a poor general physical condition. The patient cooperated well, but was only able to lie, not sit. A moderate yellowish discoloration of the patient’s skin and sclera was observed. The right inferior pulmonary percussion result was dull. Auscultation of the bronchovesicular breath sound was weak, and moist and dry rales, as well as pleural friction rub sounds, were not audible in both lungs. The patient’s abdomen was flat and gastroscopy showed no varicose veins in the gastrointestinal tract. The longitudinal splenectomy incision scar of the liver transplant procedure was present in the center of the upper abdomen in an ‘L’ shape, and had healed well. The patient’s abdomen was soft without tenderness, rebound pain, muscle tension or masses. The liver tissue 6 cm under the patient’s xiphoid process and 4 cm under the ribs was harder, with blunt edges, and auscultation showed a shifting dullness exited. There was no percussion pain in the region of the liver and kidneys. The patient’s gurgling sound in the belly had a frequency of four times per minute and there was no edema in the lower extremities. The left abdominal drainage tube was not blocked and the volume of pale yellow ascites drainage was 1,000–1,600 ml per day, and the urine volume was 500–600 ml per day. The patient’s routine blood examination, liver and kidney function and T/B lymphocyte subset test results are shown in Table I. The tests for tests for hepatitis and liver cancer were normal, the autoimmune hepatitis antibody spectrum for antinuclear antibody (ANA) was particle type (1:80) and the immunoglobulin G level and liver fibrosis tests were normal. Furthermore, the erythrocyte sedimentation rate (ESR), adenosine deaminase (ADA) and C-reactive protein (CRP) levels, tuberculosis anti-body (TBAB) and γ-interferon antibody release test for *Mycobacterium tuberculosis* in the ascites were all normal. The patient’s ascitic fluid was yellow, with a total cell number of 1.12×10^9^/l, and the Rivalta test was negative. The ascites biochemical test results are shown in Table I. An abdominal ultrasound B-mode scan showed that there were diffuse lesions in the graft liver, with no evident blood flow abnormalities, and that the liver was enlarged. The liver was located 4.5 cm under the ribs, and the maximum thickness of the right, left and caudate lobes was 13.60, 8.41 and 2.07 cm, respectively. There was moderate peritoneal effusion and a small amount of right-sided pleural effusion. The lung CT revealed encapsulated effusion in the right side of the thorax, and there was right lower pulmonary atelectasis. The abdominal CT and CTA scan results showed that the liver volume had increased and that stenosis was present in the hepatic artery anastomosis. The test results of the liver biopsy (Fig. 2) showed that the hepatic vein was occluded, and that there was necrosis with blood stasis in the center of the liver, combined with chronic cholangitis.

### Treatment process

The patient was treated with methylprednisolone (500 mg/day) as a pulse treatment for two days, with adjuvant antisecretory, gastric mucosa-protective, antibiotic and antiviral (ganciclovir) therapies. When the patient began vomiting on the third day, the pulse treatment was stopped and changed to oral Medrol (Methylprednisolone; 28 mg/day), while the tacrolimus (Prograf) antirejection therapy was stopped and changed to sirolimus tablets (2 mg/day). However, the routine blood examination results for hemoglobin (HGB) decreased significantly (ranging from 80 to 85 g/l), which resulted in the antirejection treatment being adapted with enteric-coated mycophenolate sodium tablets (540 mg/12 h, 3 weeks). In addition, the patient received fluid infusion and treatments to protect the liver, lower transaminase levels, remove jaundice and dieresis, increase ALB and improve the microcirculation of the liver [2 ml/day Prostaglandin E1 injection and 50 ml/day Danhong injection]. The liver gradually reduced in size and was located 2 cm under the xiphoid process and 2 cm under the ribs. The peritoneal drainage volume decreased gradually to 800–1,000 ml/day, the urine volume increased and liver and renal function improved gradually. The liver and kidney function examination results on the patient’s 40th day of hospital treatment (Table I) showed that the levels of ALT, AST, TBIL, B macroglobulin, DBIL, urea, UA and Cr had decreased markedly, while the level of CHE had increased. The results of the abdominal ultrasound B-mode scan showed that no significant ultrasonographical or blood abnormalities existed in the graft liver (Fig. 2). The maximum thickness of right, left and caudate lobes was 11.25, 7.84 and 1.69 cm, respectively.

The general condition of the patient improved markedly and an increase in appetite, weight gain and the ability to walk 2–3 km were observed. The patient underwent a transjugular intrahepatic portosystemic shunt (TIPS) procedure on June 18, 2011 (day 66 of hospital treatment), from which he recovered well. One week later, the patient’s ascites had disappeared and his liver and renal function had returned to normal.

## Discussion

Medication may lead to the development of VOD following liver transplantation. Izaki *et al*(8) described a number of cases of VOD following liver transplantation; however, the specific mechanism has not been elucidated. This, in combination with other studies (9,10), suggests that liver transplantation may induce postoperative VOD. Previous studies have revealed that the use of the immunosuppressant azathioprine following transplantation may damage the end of the small hepatic vein, the hepatic sinusoidal endothelial cells and the liver cells of the three zones of the hepatic lobules, causing multi-factorial abnormal pathophysiological processes, such as immune and inflammatory reactions and coagulation (11,12), leading to VOD. In the present case, the patient was treated with long-term tacrolimus following the liver transplantation, without any other drugs; we hypothesized that tacrolimus may have been the cause of the VOD. Since there have been no relevant reports, the mechanism remains to be elucidated.

The typical symptoms of VOD following liver transplantation include hepatomegaly, tenderness, weight gain, peripheral edema, ascites and jaundice. Prior to the onset of these symptoms, the patient may have had a history of HSCT, chemotherapy, radiotherapy or liver transplantation, and a long history of drinking or eating substances containing monocrotaline toxin, including tea beverages, health products, food and herbal or certain special medicines. There are two standard diagnostic references for VOD prior to HSCT and 20 days subsequent to transplantation. One is the Baltimore VOD diagnostic criteria (13), which comprises TBIL ≥34 μmol/l, combined with at least two of the following clinical manifestations: hepatomegaly accompanied by right upper abdominal pain, ascites and weight gain ≥5%. The other is the Seattle VOD diagnostic criteria (14), which includes the presence of at least two of the following manifestation and symptoms: TBIL ≥34 μmol/l, hepatomegaly, pain in the right upper quadrant or over the liver, and weight gain >20% compared with the baseline weight. In the present case, the main clinical manifestations of the patient were jaundice, hepatomegaly and pain over the liver and ascites, which were consistent with the previously mentioned diagnostic standards for VOD.

With regard to the auxiliary examinations for VOD, liver and kidney function examinations have revealed that, in cases of mild VOD, patients exhibit a mildly elevated TBIL level, while in patients with moderate and serious VOD, the TBIL level increases to 143 and 615 μmol/l, respectively; ALT and ALP levels increase concurrently (15). In addition, N-terminal domain type III procollagen peptide levels have been used in the diagnosis of VOD (16), with the majority of patients with VOD exhibiting levels of >100 μg/l. Following HSCT, patients’ CRP levels often decrease prior to the development of VOD, showing a predictive value for VOD of 91%, a specificity of 87% and a sensitivity of 69% (15). In addition, plasminogen activator agent inhibitor-1 (PAI-1) levels have been indicated to be a diagnostic marker of VOD following HSCT, with levels increasing in the early stages of VOD (17). With regard to imaging examinations, color Doppler ultrasound is of no use in the diagnosis of VOD. However, the measurement of wedged hepatic vein pressure and hepatic venous pressure gradient (HVPG) using an intravenous cannula may identify whether portal hypertension is a result of VOD, with HVPG >10 mmHg prompting a diagnosis of VOD (18). Furthermore, irregular small hepatic vein waves and patchy contrast medium filling the liver parenchyma in hepatic venography are imaging results that are indicative of VOD. In addition to the previously mentioned diagnostic methods, an abdominal CT of patients with VOD typically shows an increased liver volume and congestion of the liver; however, liver vascular remodeling does not support the stenosis of the hepatic vein and inferior vena cava, which is one differentiating factor from Budd-Chiari syndrome.

In conclusion, the pathophysiology of VOD has not been fully elucidated. As an increasing number of cases of patients with VOD following liver transplantation are investigated, it may be possible to obtain an enhanced understanding of the mechanism underlying the disease.

## Figures and Tables

**Table I:** Patient examination results.

	Level or concentration	
	
Examination	Admission	40th day of hospital treatment
Routine blood examination		
WBC (per liter)	12.67×10^9^	-
NEUT (%)	41.2	-
HGB (g/l)	110	-
PLT (per liter)	270×10^9^	-
Liver and kidney function		
ALT (IU/l)	56	29
AST (IU/l)	170	31
GGT (IU/l)	-	184
ALP (IU/l)	-	91
TBIL (μmol/l)	235.1	23.3
DBIL (μmol/l)	169.6	141.0×10^4^
ALB (g/l)	30.1	31.6
CHE (IU/l)	1132	2676
B macroglobulin (mg/l)	17.42	4.26
Urea (mmol/l)	31.68	19.59
UA (μmol/l)	828	529
Cr (μmol/l)	178	81
T/B lymphocyte subsets		
CD4 (cells/μl)	1485	-
FK (ng/ml)	5068.5	-
Ascites biochemical tests		
Glu (mmol/l)	7.04	-
LDH (U/l)	68	-
TP (g/l)	29.8	-
ADA (IU/l)	6	-
ALB (g/l)	11.9	-

WBC, white blood cells, NEUT, neutrophils; HGB, hemoglobin; PLT, platelets; ALT, alanine transaminase; AST, aspartate transaminase; GGT, γ-glutamyl transpeptidase; ALP, alkaline phosphatase; TBIL, total bilirubin; DBIL, direct bilirubin; ALB, albumin; CHE, cholinesterase; UA, uric acid; Cr, creatinine; Glu, glucose; LDH, lactate dehydrogenase; TP, total protein; ADA, adenosine deaminase.
